# SURVEY OF CHANGES IN SUBJECTIVE SYMPTOMS AMONG JAPANESE POLIO SURVIVORS OVER 10 YEARS

**DOI:** 10.2340/jrm.v57.42213

**Published:** 2025-04-22

**Authors:** Fumi TODA, Koshiro SAWADA, Daisuke IMOTO, Kazuya HAYASHI, Shun FUJII, Eiichi SAITOH, Yohei OTAKA

**Affiliations:** 1 Department of Rehabilitation Medicine, School of Medicine, Fujita Health University, Aichi; 2 Department of Rehabilitation Medicine, Kyoto Prefectural University of Medicine, Kyoto; 3 Department of Rehabilitation, Fujita Health University Hospital, Aichi, Japan

**Keywords:** muscle, weakness, atrophy, poliomyelitis, survey, symptom, limb

## Abstract

**Objective:**

To define long-term changes in subjective symptoms among polio survivors in Japan.

**Design:**

Prospective cohort study.

**Patients:**

Sixty-five polio survivors.

**Methods:**

Surveys were conducted on subjective symptoms including muscle weakness and limb atrophy during 2007 and 2021. The results of manual muscle tests of the upper and lower limbs on both sides during 2007 were summed and scored, and the side with lower scores was defined as the poor side. The participants were classified as younger or older groups based on the median age at the first survey (i.e., 58 years old) and the subjective symptoms were compared between the two groups.

**Results:**

As a whole, muscle atrophy and weakness progressed in the lower and upper limbs while fatigue was reduced. Muscle weakness progressed especially in the lower limbs on the poor side in the younger group, and in the older group it progressed in the lower limbs on the good side and the upper limbs on the poor side.

**Conclusion:**

The timing of progressive muscle weakness differed between the upper and lower limbs of younger and older polio survivors.

## LAY ABSTRACT

Poliomyelitis causes muscle weakness, often in good and poor limbs. Although polio survivors frequently develop post-poliomyelitis syndrome that comprises new muscle weakness, long-term changes remain unclear. We investigated changes in subjective symptoms, especially muscle weakness on the good and poor sides, in 65 Japanese polio survivors with a median age of 58 years at the time of diagnosis over > 10 years. An evaluation of degrees of muscle weakness in the upper and lower limbs and symptoms revealed increased muscle weakness and atrophy. Muscle weakness was progressive in the poor lower limbs of younger survivors and in the good lower limbs and poor upper limbs of older survivors. The timing of progressive muscle weakness differed between the upper and lower limbs of younger and older polio survivors.

Poliomyelitis is a paralysing infectious disease that affects infants and young children, which became a global pandemic during the 20th century. However, routine vaccination was introduced worldwide during the 1970s, which resulted in epidemics occurring in only Pakistan and Afghanistan (1). However, many polio survivors experience post-polio syndrome (PPS), which presents as new muscle weakness, decreased muscle endurance, muscle atrophy and pain, joint pain, generalized fatigue, breathing and swallowing problems, decreased function, and cold intolerance (2, 3). The pathogenesis of PPS is thought to involve overuse or premature ageing of polio-affected motor units (4). Therefore, symptoms that manifest in polio survivors are considered progressive and should be followed up for extended periods.

Various health problems and new PPS-related subjective symptoms develop after 10 years or more (5, 6). A Swedish study reported that the proportion of patients with symptoms of muscle weakness, fatigue, and atrophy did not change over a period of 17 years, although many participants had such symptoms (5). In contrast, a Norwegian study reported that the proportion of polio survivors with subjective symptoms of muscle weakness, atrophy, muscle pain, joint pain, cold intolerance, insomnia, and lower-limb swelling increased, whereas those of fatigue decreased over a period of 20 years (6). These studies investigated subjective muscle weakness in limbs affected and unaffected by poliomyelitis and did not reach consensus in terms of changes in subjective muscle strength (5, 6). This might have been influenced by the timing of surveys and the characteristics of the survivors, such as age. Furthermore, although polio survivors have muscle weakness in different limbs due to poliomyelitis (7, 8), their limbs could be more appropriately classified as good or poor side to understand muscle weakness. Therefore, the evidence of long-term changes in subjective symptoms among polio survivors is inconsistent and requires further investigation.

However, previous cohort studies have been conducted mainly in Europe (5, 6), and long-term changes in PPS-related symptoms have not been investigated in Asian polio survivors. A major polio epidemic in Japan during 1960 affected > 5,000 people, which subsided rapidly after a vaccine was introduced (9). A nationwide survey has not been conducted in Japan, but a cross-sectional study in Kitakyushu identified a PPS prevalence of 18.0 per 100,000 polio survivors (10). Therefore, it is important to improve understanding of long-term changes in subjective symptoms among Japanese polio survivors, and for polio survivors to participate in comprehensive rehabilitation programmes.

This study aimed to define long-term changes in subjective symptoms associated with PPS in Japanese polio survivors. We tested the hypothesis that long-term PPS-associated symptoms such as muscle weakness, muscle atrophy, and fatigue would worsen; changes in subjective muscle weakness in the upper and lower limbs, or on the good or poor sides would differ; and such changes would differ according to age, which is an important factor associated with PPS (11, 12).

## METHODS

### Ethics

The Medical Research Ethics Review Committee at Fujita Health University (Approval ID: HM15-117) approved this prospective cohort study, which complied with ethical principles outlined in the Declaration of Helsinki (2013 amendment). The results were reported according to the Strengthening Reporting of Observational Studies in Epidemiology (STROBE) guidelines (13). All participants provided written informed consent to participate in the study.

### Participants

We established a collaboration with a support group for polio survivors in the Tokai area of Japan to survey and periodically monitor polio survivors during 2006. The association comprises polio survivors residing in the Aichi, Gifu, and Mie prefectures. We conducted the first survey with a questionnaire sent to 181 association members during 2007 and received responses from all of them (first survey). From the first to the second survey, 86 individuals dropped out of the support group. Therefore, the second questionnaire was mailed to 95 individuals in June 2021 (second survey). As a result, we received responses from 65 individuals who were included in the analysis.

### Survey

We used questionnaires to collect data on demographic characteristics, age at the time of poliomyelitis onset and when they responded to surveys, sex, subjective symptoms, walking aid use, orthosis use, and follow-up at the clinic (only in the second survey). The distribution of paralysis was determined on both, 1, or no sides in the upper and lower limbs at the time of poliomyelitis onset and when the survivors responded to the first survey. Data concerning the following PPS-associated symptoms (2, 3) were collected from both surveys: muscle atrophy, gait disturbance, fatigue, coldness, functional impairment, joint and muscle pain, shortness of breath, and swallowing difficulty. The severity of muscle weakness in each limb was graded as none, mild, severe, and immobile.

### Diagnosis of PPS and determination of the good and poor sides of limbs

The diagnosis of PPS and determining the good and poor sides of limbs were based on the findings of the first survey that took place at the outpatient clinic of the department of rehabilitation medicine at Fujita Health University Hospital. The patient was diagnosed with PPS by a physiatrist who examined the patient based on the Halstead diagnostic criteria (3). When PPS could not be confirmed, the participant was considered not to have PPS. Physical and occupational therapists determined the good and poor sides of the upper and lower limbs using manual muscle tests (MMT) (14). For the lower limbs, the MMT scores of hip flexion, extension, and abduction, knee extension and flexion, ankle dorsal, and plantar flexion were summed on each side, and the side with a lower total score was defined as the poor side. The MMT scores of shoulder abduction, elbow flexion, and extension were summed for the upper limbs on both sides, and a lower total score was considered to indicate the poor side.

### Statistical analysis

Interval and ordinal variables are presented as medians and interquartile ranges, whereas categorical variables are presented as numbers and ratios (%). The severity of muscle weakness was graded as 0 (none), 1 (mild), 2 (severe), or 3 (immobile). Participants with missing data were excluded from analysis. Changes in prevalence of subjective symptoms were compared between the first and second surveys using McNemar tests. Changes in the severity of muscle weakness in the upper and lower limbs were compared between the first and second surveys using Wilcoxon signed-rank tests.

Ageing is a risk factor for PPS (11, 12). Therefore, we assigned the participants to younger and older groups based on their median age at the time of the first survey and compared changes in subjective symptoms between the two surveys using McNemar tests. Changes in the severity of muscle weakness in the upper and lower limbs and the good and poor sides between the 2 surveys were investigated using Wilcoxon signed-rank tests. For analysis of the comparison between good and poor sides, participants with equal total MMT scores on both sides were excluded from the analysis. All data were statistically analysed using IBM SPSS Statistics version 23.0 (IBM Corp, Armonk, NY, USA) and significance was set at *p* < 0.05.

## RESULTS

**Fig. 1:**
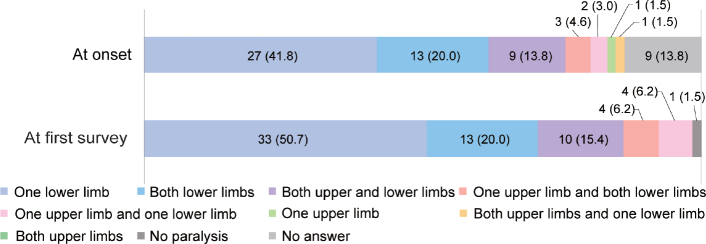
Changes in distribution of paralysis in upper and lower limbs at onset and first survey. Data are presented as numbers of participants (%).

The participants’ characteristics in this study are presented in Table I. The median (minimum–maximum) follow-up period of the 65 polio survivors who responded to both surveys was 14 (13–14) years. Among the 65 participants, 45 (69.2%) had PPS. Most of those not diagnosed with PPS (20, 30.8%) were not diagnosed because they did not meet one of the Halstead diagnostic criteria regarding new symptoms (criteria require at least 2 or more symptoms such as extensive fatigue, muscle and/or joint pain, new weakness in muscles previously affected or unaffected after the stable period). All participants had no other medical, neurological, or orthopaedic conditions diagnosed based on the Halstead diagnostic criteria at the time of the first survey. Fig. 1 shows that the most prevalent type of paralysis at poliomyelitis onset and at the time of the first survey affected a single lower limb. In the first survey, the commonest type of paralysis was monoparesis in the lower limb (50.7%), and almost all except 1 participant (98.5%) had weakness in the lower limb(s), and most participants (72.2%) had no paresis in the upper limbs.

**Table I:** Participants’ characteristics

Characteristic	Overall (*n* = 65)	Younger (*n* = 28)	Older (*n* = 37)
Age years, median (IQR)			
At onset	1 (0–3)	1 (0–2.3)	1 (0–3)
At the first survey	58 (56–62)	55 (49–56.3)	60 (58–65)
At the second survey	72 (70–72)	70 (63–71)	75 (73–79)
Male, *n* (%)	18 (27.7)	6 (21.4)	12 (32.4)
Post-polio syndrome at the first survey, *n* (%)	45 (69.2)	20 (71.4)	25 (67.6)
Walking aid use, *n* (%)			
At the first survey	22 (33.8)	9 (32.1)	13 (35.1)
At the second survey	56 (86.2)	26 (92.9)	30 (81.1)
Orthosis, *n* (%)			
At the first survey	33 (50.8)	15 (53.6)	18 (48.6)
At the second survey	53 (81.5)	23 (82.1)	30 (81.1)
Follow-up at clinic, *n* (%)	37 (56.9)	18 (64.3)	19 (51.4)

IQR: interquartile range.

**Fig. 2:**
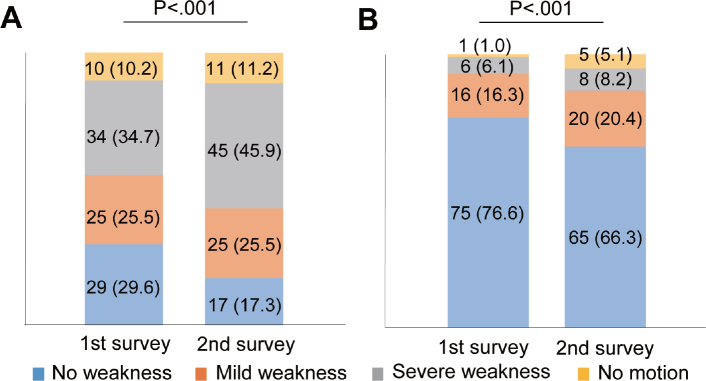
Changes in subjective muscle weakness in upper and lower limbs: analyses as a left–right sum. Bar graphs show changes in subjective muscle strength of (A) lower (*n* = 98) and (B) upper (*n* = 98) limbs. Data are presented as numbers of limbs (%). Missing data, 32 limbs in 16 participants.

**Fig. 3:**
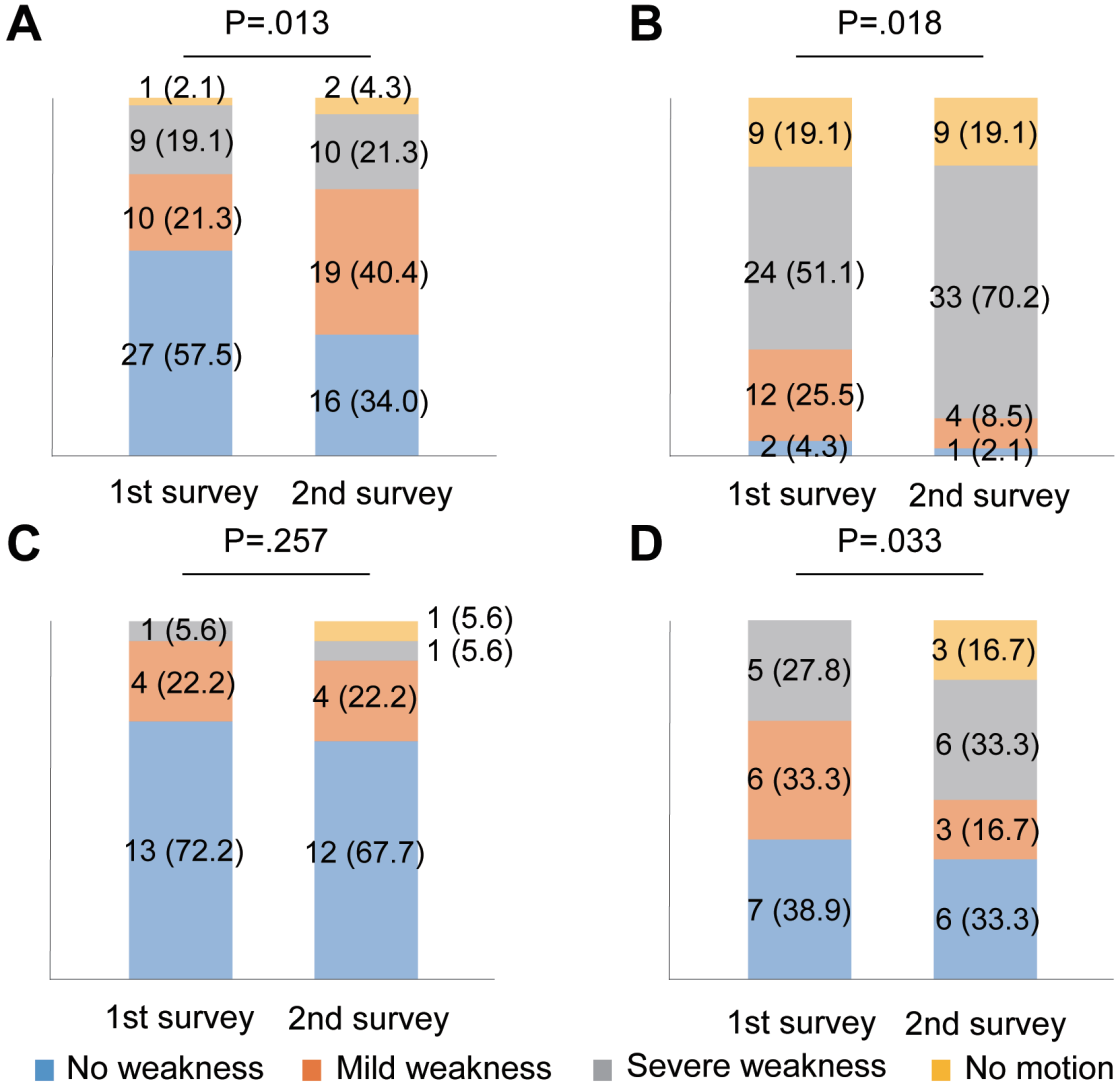
Changes in subjective muscle weakness in upper and lower limbs on good and poor sides. Bar graphs show changes in subjective muscle strength in lower limbs on (A) good (*n* = 47) and (B) poor (*n* = 47) sides, (C) upper limbs on good (*n* = 18), and (D) poor (*n* = 18) sides. Data are presented as numbers of limbs (%). Excluded data: 58 upper limbs in 29 participants due to equal muscle strength bilaterally; missing data, 36 each of upper and lower limbs in 18 participants.

The prevalence of muscle atrophy and fatigue between the first and the second surveys significantly increased (*p* < 0.001) and decreased (*p* = 0.007), respectively (Table II). No other subjective symptoms changed between these surveys. Regarding muscle weakness, the severity of muscle weakness progressed in the lower and upper limbs in analyses as a left–right sum (*p* < 0.001 for both Fig. 2A and B). When dividing each limb into the good side or poor side, the severity of muscle weakness significantly progressed on both sides of the lower (good side: *p* = 0.013, poor side: *p* = 0.018) and the poor side of the upper (*p* = 0.033) limbs (Fig. 3A, B, and D). The severity of muscle weakness did not change on the good side of the upper limbs (p = 0.257; Fig. 3C).

**Table II:** Changes in symptoms at first and second surveys

Symptom	First survey (%)	Second survey (%)	*p*-value
Muscle atrophy‡	34.5	94.8	< 0.001
Gait disturbance	95.4	87.7	0.227
Fatigue	86.2	66.2	0.007
Coldness	63.1	58.5	0.678
Functional impairment†	66.7	58.7	0.383
Joint pain	60.0	55.4	0.664
Muscle pain	43.1	53.8	0.210
Shortness of breath*	32.8	28.1	0.690
Swallowing difficulty*	12.5	12.5	> 0.999

Data missing for *1, †2, and ‡7 cases.

**Fig. 4:**
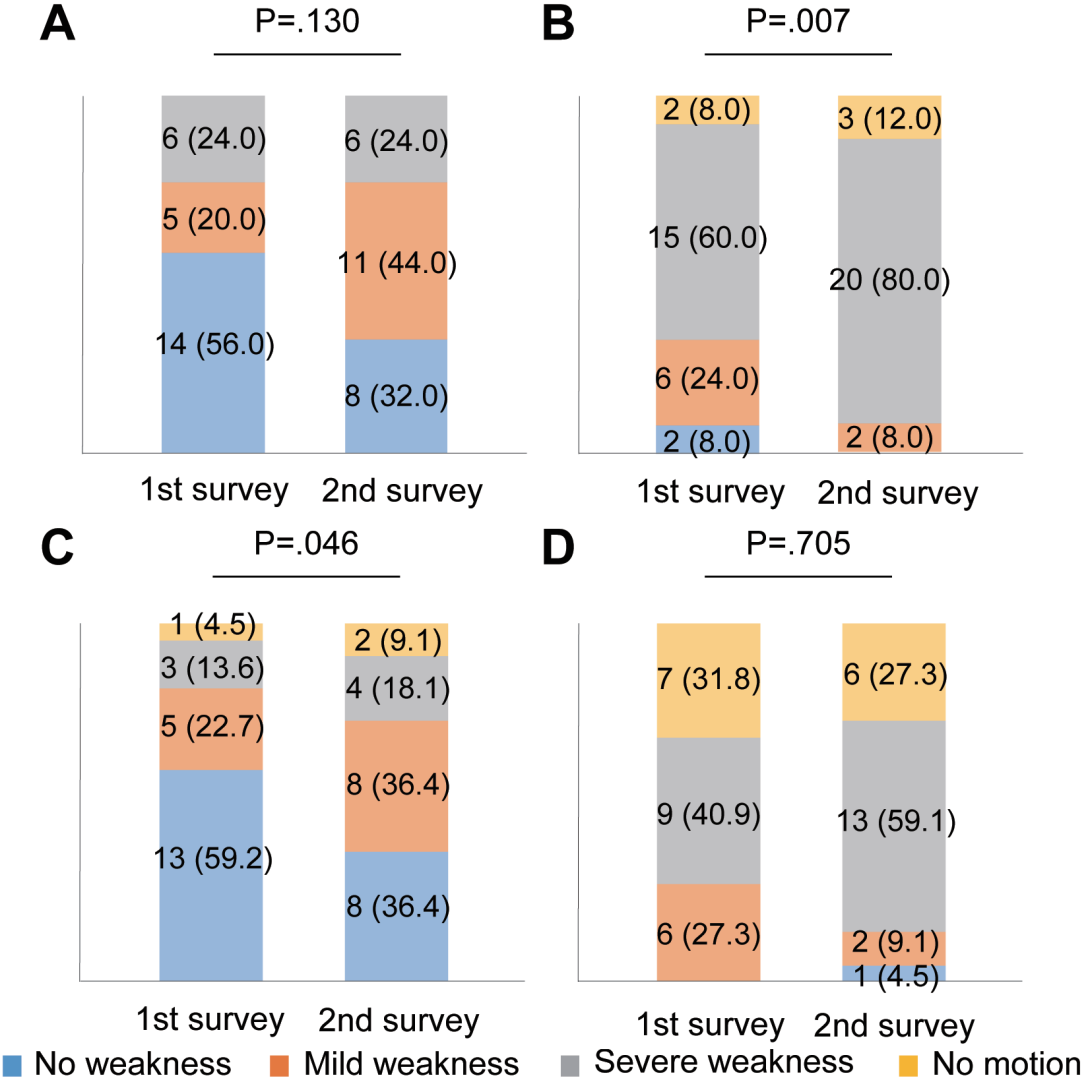
Changes in muscle weakness of lower limbs on each side in younger and older groups. Bar graphs show changes in subjective muscle strength in (A) good (*n* = 25) and (B) poor sides in the younger group (*n* = 25), (C) good (*n* = 22), and (D) poor sides in the older group (*n* = 22). Data are presented as numbers of limbs (%). Missing data, 36 limbs in 18 participants.

**Fig 5:**
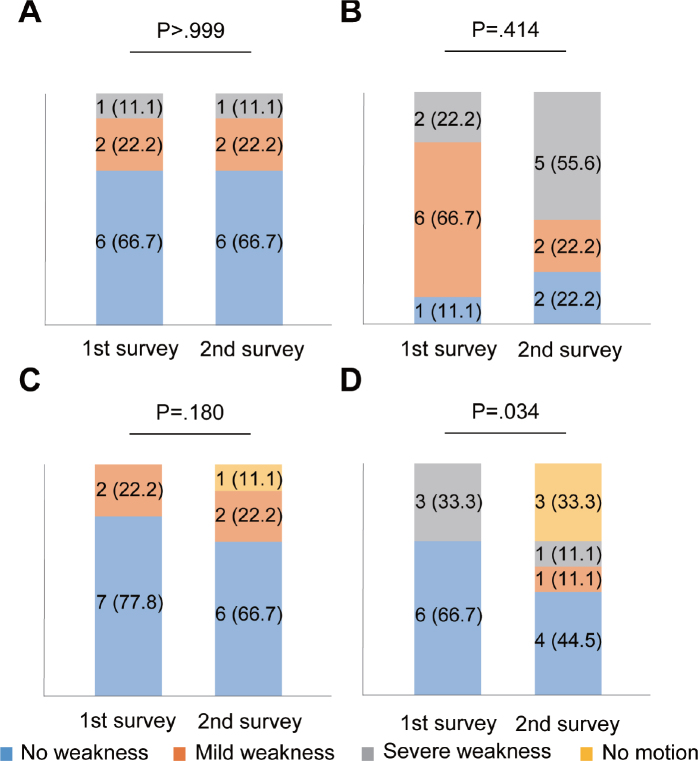
Changes in muscle weakness of upper limbs on each side in younger and older groups. Bar graphs show changes in subjective muscle strength in (A) good (*n* = 9) and (B) poor sides in the younger group (*n* = 9), (C) good (*n* = 9), and (D) poor sides in the older group (*n* = 9). Data are presented as numbers of limbs (%). Excluded data: 58 limbs in 29 participants due to equal muscle strength bilaterally; missing data, 36 limbs in 18 participants.

As subgroup analysis, based on the median age of the participants (58 years), participants were divided into 28 participants (43.1%) in the younger group and 37 (56.9%) in the older group. The prevalence of muscle atrophy increased (*p* < 0.001) and fatigue decreased (*p* = 0.011) in the younger group, whereas that of muscle atrophy increased (*p* < 0.001) in the older group between the first and second surveys (Table III). The severity of muscle weakness worsened on the poor side of lower limbs (*p* = 0.007; Fig. 4B), but did not change on the good side (*p* = 0.130; Fig. 4A) in the younger group. In contrast, the severity of muscle weakness worsened on the good side (*p* = 0.046; Fig. 4C) but did not change on the poor side (*p* = 0.705; Fig. 4D) in the older group. The severity of upper-limb muscle weakness did not worsen on both sides in the younger group, respectively (*p* > 0.999 and p = 0.414; Fig. 5A and B). The severity of muscle weakness did not significantly change on the good side (*p* = 0.180; Fig. 5C), but significantly worsened on the poor side of the upper limbs (*p* = 0.034) in the older group (Fig. 5D).

**Table III:** Changes in symptoms between first and second surveys in younger and older groups

Symptom	Younger (*n* = 28)			Older (*n* = 37)		
	First survey (%)	Second survey (%)	*p*-value	First survey (%)	Second survey (%)	*p*-value
Muscle atrophy	36.0†	96.4†	< 0.001	33.3‡	94.6‡	< 0.001
Gait disturbance	96.4	89.3	0.317	94.6	86.5	0.257
Fatigue	85.7	57.1	0.011	86.5	73.0	0.132
Coldness	60.7	60.7	> 0.999	64.9	56.8	0.366
Functional impairment	55.6*	60.7*	0.739	75.0*	59.5*	0.083
Joint pain	64.3	64.3	> 0.999	56.8	48.6	0.317
Muscle pain	50.0	57.1	0.527	37.8	51.4	0.166
Shortness of breath	33.3*	25.0*	0.564	32.4	29.7	0.782
Swallowing difficulty	14.3	14.3	> 0.999	11.1*	10.8*	> 0.999

Data missing for *1, †3, and ‡4 cases.

## DISCUSSION

We define changes in subjective symptoms among 65 Japanese polio survivors for > 10 years. The prevalence of muscle atrophy was found to increase, whereas that of fatigue decreased. As for the age difference, atrophy increased in both younger and older groups, while fatigue decreased only in the younger group. Muscle weakness progressed on both sides of the lower and the poor side of the upper limbs as a whole. Muscle weakness worsened in the lower limbs on the poor side in the younger group. In the older group, muscle weakness progressed in the lower limbs on the good side and the upper limbs on the poor side. Except for the findings in fatigue, the findings obtained in the present study supported our hypothesis that symptoms would worsen and change in muscle weakness in the upper and lower limbs, or on the good or poor sides would differ, and such changes would also differ according to age.

As for the muscle atrophy and weakness, our findings of increased subjective muscle weakness and muscle atrophy were consistent with those of a Norwegian cohort that was followed up for 20 years (6). We believe that the reason for this is the close match between the characteristics of the participants in the previous study and ours. In contrast, our results differed from those of a Swedish cohort study, which reported that the proportion of patients with symptoms of muscle weakness and atrophy did not change over a period of 17 years (5). One possible reason is the difference in age at the onset of polio between the previous study and ours. Risk factors for symptoms and functional decline for polio survivors were reported as age at polio onset and the length from polio onset (11). The median age at onset of polio was 1 year old in the present study and 10 years old in the Swedish study (5), respectively. However, the median age of the participants at the time of the follow-up survey was almost the same in both studies: 72 and 70 years old in the present study and in the Swedish study, respectively. In other words, these results show that the participants in our study had a longer time from the onset of polio than the participants in the previous study. Therefore, we believe that the difference in duration from the onset of polio caused differences in long-term changes in symptoms.

The strength of this study is that we separately clarified the progression of subjective muscle weakness in both poor and good lower and upper limbs. A study showing longitudinal changes in muscle strength using 30 upper and lower extremity movements found significantly reduced muscle strength in the lower and upper extremities (15). However, the characteristics of muscle weakness progression have not been compared between poor and good limbs as far as we can ascertain. The present study revealed progressive muscle weakness, especially in the lower limbs on both sides and in the upper limbs on the poor side. We believe that this finding is due to overuse in the muscles. Overuse is one reason for progressive muscle weakness among polio survivors (16, 17). For example, unilateral or bilateral quadriceps and hip extensors are overused to compensate for weak calf muscles (18). In the present study, most participants had paresis in the lower limb(s), which conferred additional stress in both lower limbs that led to overuse. Furthermore, a study showed that muscle weakness in the lower limbs influences the development of symptoms due to overuse in the upper limbs (19). The present study revealed muscle weakness in the upper limbs, especially on the weaker side. We believe that the muscle weakness in the lower limb may have led to compensatory movement in the lower limbs and the upper limbs, resulting in muscle weakness in these limbs. Therefore, our findings suggested that the management of muscle weakness in polio survivors requires consideration of its effects on the lower and upper limbs.

Regarding the age difference in muscle weakness, muscle weakness increased in the lower limbs on the poor side in the younger group, while muscle weakness progressed in the lower limbs on the good side and the upper limbs on the poor side in the older group. These age differences can be explained, at least in part, by age-related muscle loss in the limbs as follows. A study has reported that the reduction in muscle mass related to ageing was greater in the lower limbs than in the upper limbs (20). This is probably the reason why muscle weakness progressed even in the lower limbs on the good side in the older group. Also, the younger group did not perceive any muscle weakness in the upper limbs. Importantly, this study found that lower limb muscle weakness affected approximately 40% of participants, even on the good side (Fig. 3A). A cohort study among Japanese older adults reported that the prevalence of sarcopenia, a symptom of muscle weakness, was 11.5% in men and 16.7% in women (21). Thus, a much higher percentage of polio survivors were aware of muscle weakness. We believe that polio survivors have a higher risk of muscle weakness due to the combination of compensatory overuse of the limbs and age-related muscle weakness compared with older adults living in the community.

Notably, fatigue was reduced, especially in the younger group, in contrast to muscle atrophy and muscle weakness. The median age of the younger group was 55 years in the first survey and 70 years in the second survey. At this age, lifestyles can change significantly (e.g., retirement usually occurs around age 60 in Japan). Age-related lifestyle changes may have influenced the decrease in activity, leading to a decrease in subjective fatigue.

This study has several limitations. We determined symptoms using a subjective scale. Therefore, we could not define whether the participants had actual functional abnormalities. Future investigations should use more objective means to sensitively detect changes in symptoms associated with PPS. This study had the potential for selection bias. The support group for polio survivors in the Tokai area of Japan is a small regional group of polio survivors. Other polio survivors might exist who have not joined patient associations because they do not have symptoms associated with PPS. Moreover, the participants were screened in a hospital outpatient setting, thus excluding polio survivors who were too ill to attend the hospital. Therefore, our results should be interpreted with caution before they can be generalized.

In conclusion, prevalence of muscle weakness and atrophy increased, whereas that of fatigue decreased in polio survivors. Furthermore, the timing of progressive muscle weakness differed between the upper and lower limbs. This suggests that which limbs will develop muscle-associated symptoms might become predictable in the future. Meanwhile, polio survivors should be cautioned to avoid overuse of limbs by using orthotics, canes, crutches, and to seek advice regarding activities of daily living when anticipating new muscle-associated symptoms.
